# Reporting of Y Balance Test Measurement Procedures in Reliability and Validity Studies: A Scoping Review

**DOI:** 10.3390/sports14050191

**Published:** 2026-05-06

**Authors:** Hiroto Takahashi, Tatsuya Igawa, Ryunosuke Urata, Shomaru Ito, Kosuke Suzuki, Riyaka Ito, Mika Toda, Chiaki Matsumoto, Masahiro Ishizaka

**Affiliations:** 1 Department of Physical Therapy, Graduate School of International University of Health and Welfare, 2600-1 Kitakanemaru, Ohtawara 324-8501, Japan; hiro9taka6@gmail.com (H.T.); riyakaito@gmail.com (R.I.); 2 Department of Physical Therapy, School of Health Sciences, International University of Health and Welfare, 2600-1 Kitakanemaru, Ohtawara 324-8501, Japan; m-chiaki@ihwg.jp (C.M.); ishizaka@ihwg.jp (M.I.); 3 Innovative-Rehabilitation Center, New Spine Clinic Tokyo, 2-6-3 Hirakawacho, Chiyoda-ku, Tokyo 102-0093, Japan; ryu.urata62@gmail.com; 4 Department of Physical Therapy, School of Health Sciences at Odawara, International University of Health and Welfare, 1-6 Minamicho, Odawara 250-0013, Japan; shomaru.ito5151@gmail.com; 5 Department of Rehabilitation, Yamagata Saisei Hospital, 79-1 Okimachi, Yamagata 990-0818, Japan; kousuke.s0922@gmail.com; 6 Department of Rehabilitation, International University of Health and Welfare Nasu Medical Center, 573-3 Iguchi, Nasushiobara 329-2763, Japan; todamika2@gmail.com

**Keywords:** Y Balance Test, reliability and validity, reporting quality of measurement procedures

## Abstract

The Y Balance Test (YBT) is widely used to assess dynamic balance and lower-limb function in both clinical and sports settings. However, substantial variability exists in YBT measurement procedures across studies, which may complicate interpretation of the reliability and validity findings. This scoping review aimed to systematically map the reporting frequency and heterogeneity of participant characteristics and measurement procedures in studies evaluating the reliability and/or validity of the YBT. This scoping review was conducted in accordance with the PRISMA extension for Scoping Reviews and the Joanna Briggs Institute methodology. MEDLINE, CENTRAL, CINAHL, and clinical trial registries were searched from inception to 20 September 2025. Observational studies assessing the reliability and/or validity of the YBT were also included. Data on participant attributes and key measurement procedures, including practice trials, rest periods, upper-limb restrictions, heel lift allowance, and other protocol components, were extracted. A total of 32 studies involving 1701 participants were analyzed. Considerable heterogeneity was observed in the reporting of YBT measurement procedures across studies, with differences according to study design and participant characteristics. While practice trials and error criteria were frequently described, other factors that may influence outcomes, including warm-up protocols, rest periods, heel lift allowance, and trial order, were less consistently reported. Such variability may limit the interpretability and comparability of YBT findings. Notable gaps were identified in both reliability and validity studies, as well as in studies involving injured or clinical populations. These findings highlight the need for greater transparency and consistency in YBT reporting. Clearer reporting standards may improve evidence synthesis and the application of YBT findings.

## 1. Introduction

Balance is a multifaceted capability that arises from the integrated interaction of sensory, neurological, musculoskeletal, and cognitive systems and serves as a fundamental prerequisite for a broad spectrum of physical activities, ranging from activities of daily living to high-level competitive sports [1]. Accordingly, the assessment of dynamic balance has attracted growing attention in both clinical and sports science contexts, given its close association with injury risk reduction and optimization of physical performance [2].

The Y Balance Test (YBT) is extensively employed as a practical and efficient instrument for the assessment of lower-limb function and dynamic balance, with broad applications that include return-to-sport decision-making, evaluation of lower-limb injury risk, and prediction of fall risk among older adults [3]. The YBT protocol was initially proposed by Plisky et al. [4]. However, considerable variability in YBT measurement protocols has been documented in the literature [5]. In particular, differences have been reported with respect to warm-up procedures, number of practice trials, upper-limb positioning, heel lift allowance, and the number of test trials administered. Such methodological heterogeneity has been suggested to potentially affect the reliability and validity of YBT outcomes [6]. Greenberg et al. highlighted the importance of adopting measurement protocols that account for physical growth and age in a cohort of adolescent female athletes [7]. In recent years, a growing body of research has investigated participant characteristics along with the reliability and validity of the YBT across diverse populations, a trend that may represent a key source of heterogeneity in YBT measurement methods. Consequently, YBT measurement procedures should be systematically organized with a primary emphasis on evidence derived from reliability and validity research. Nevertheless, to the best of our knowledge, no scoping review has comprehensively addressed this issue. It is important to note that measurement procedures can affect comparability in studies examining the reliability and validity of YBT. Therefore, careful attention to these procedures is required. However, existing reviews have not specifically focused on the reliability and validity of participant characteristics or measurement procedures.

Previous reviews primarily summarized reliability according to reach directions and sport-specific trends [8]. However, no studies have systematically investigated the differences in participant characteristics or measurement procedures, specifically within the framework of reliability and validity. The findings of this review contribute to the potential standardization of YBT measurement methods by explicitly accounting for variability in participant attributes and study design. Accordingly, this review offers both researchers and clinicians a clearer conceptualization of methodological heterogeneity while facilitating efficient access to studies supported by established evidence of reliability and validity.

Based on these considerations, the purpose of this study was to systematically identify and analyze the current state of reporting regarding participant characteristics and the YBT measurement procedures in studies evaluating the reliability and validity of the YBT. In this study, we classified the commonly reported YBT procedures according to the frequency of reporting for each item and identified inconsistencies in their descriptions. The findings of this study may assist researchers and clinicians in selecting appropriate YBT measurement procedures based on adequately reported information.

## 2. Materials and Methods

This study was conducted as a scoping review in accordance with the Preferred Reporting Items for Systematic Reviews and Meta-Analyses (PRISMA) extension for Scoping Reviews and Joanna Briggs Institute methodology [9,10] (Supplementary Table S1). The study protocol was prospectively registered using the Open Science Framework https://osf.io/t2e8f/overview (accessed on 26 September 2025). The literature search included observational studies that investigated the reliability (intra-, inter-, and test–retest reliabilities) and validity (criterion-related and construct validity) of the YBT, including cross-sectional, cohort, and case–control designs. In the included studies, participant characteristics such as age group, sport, competitive level, and injury status were required to be clearly reported. Details regarding measurement procedures in reliability and validity studies were not included in the eligibility criteria and were not used for screening. The presence or absence of these items was assessed during the data extraction phase. No restrictions were imposed on sex, age, or language. Studies were excluded if they were intervention studies, case series, review articles, or conference abstracts; if the full text was unavailable; or if the YBT was used solely as a reference measure for the validation of other assessment tools. Additionally, studies that lacked sufficient descriptions of participant characteristics were excluded as they did not align with the primary aim of this review, which was to clarify the relationship between participant characteristics and YBT measurement procedures.

Literature searches were performed across three electronic databases: MEDLINE (via PubMed), Cochrane Central Register of Controlled Trials, and Cumulative Index to Nursing and Allied Health Literature via EBSCOhost. Clinical trial registries were searched using the World Health Organization International Clinical Trials Registry Platform and ClinicalTrials.gov. Medical Subject Headings and relevant keywords (e.g., “Y Balance Test,” “YBT,” “reliability,” “validity,” and “dynamic balance”) were combined, with search strategies tailored to the specific requirements of each database. The full search strategy was presented in the Supplementary Materials (Supplementary Table S2). The final search was conducted on 20 September 2025. All retrieved records were imported into Rayyan https://www.rayyan.ai/ (accessed on 26 September 2025) and duplicate entries were identified and removed using both automated and manual procedures. To ensure comprehensiveness, additional searches were conducted to identify theses and other relevant items of gray literature. The gray literature identified during the search process was excluded in accordance with predetermined selection criteria. When full-text articles were not accessible from databases or registries, the corresponding authors were contacted directly to obtain the necessary information.

Study screening was conducted independently by two reviewers who first evaluated titles and abstracts and subsequently assessed full-text articles to determine eligibility. Discrepancies were resolved through discussion and consultation with a third reviewer (T.I.) when necessary. The reasons for exclusion at the full-text screening stage were systematically recorded and presented in the PRISMA flow diagram.

Extracted data included study design, participant characteristics (e.g., age, sex, and athletic level), and detailed descriptions of the YBT measurement procedures. Based on previous reviews [11], the extracted procedural elements included the presence of a warm-up, presence and number of practice trials, upper-limb restrictions, barefoot testing, allowance of heel lift, specification of trial direction order, predefined error criteria for failed trials, inclusion of rest periods, use of a YBT kit, normalization of outcome measures, and number of test trials. As this study constituted a scoping review of the existing literature and did not involve new data collection from human participants, ethical approval was not required.

## 3. Results

**Figure 1:**
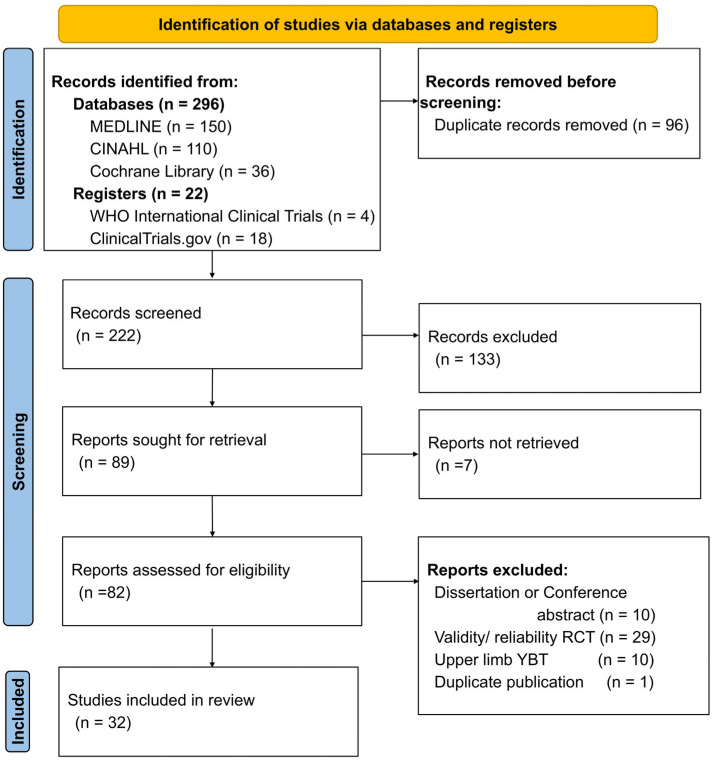
PRISMA flow chart with information on the specifics of the search process.

The database and clinical trial registry searches yielded 318 records. After duplicate entries were removed, 222 articles underwent title and abstract screening. Of these, 89 articles were deemed potentially eligible and assessed for full-text availability. Full texts were successfully retrieved from 82 articles that were subsequently evaluated through full-text screening. Finally, 32 studies satisfied the eligibility criteria and were included in this review (Figure 1).

The analysis included 32 studies encompassing a total of 1701 participants [3,4,5,7,12,13,14,15,16,17,18,19,20,21,22,23,24,25,26,27,28,29,30,31,32,33,34,35,36,37,38,39]. Among these, 21 studies exclusively investigated reliability [3,4,5,7,12,15,16,17,18,21,22,25,26,28,32,33,34,36,37,38,39], five studies focused solely on validity [14,20,30,31,39], and six studies examined both reliability and validity [13,19,20,24,27,35]. Based on reported participant characteristics, 12 studies classified participants as healthy individuals (e.g., young healthy adults or recreational-level participants) [3,5,12,20,21,22,24,26,29,31,33,34], 13 studies involved athletic populations [4,5,7,14,15,16,17,18,19,23,25,28,32], and seven studies targeted injury-related groups, defined as participants with specific injury sites or injury conditions [13,27,30,35,36,38,39] (Table 1).

Marked heterogeneity was observed in the reporting of the YBT measurement procedures across the 32 included studies. Six commonly reported procedural items were systematically extracted based on the review framework and summarized across the included studies. The presence or absence of practice trials was reported in 24 studies (75.0%), and predefined error criteria for failed trials were described in 25 studies (78.1%). The use of a YBT kit was documented in 23 studies (71.9%), and the normalization of outcome measures was reported in 25 studies (78.1%). In contrast, the implementation of a pre-measurement warm-up was reported in only four studies (12.5%), and explicit permission to lift the heel off the ground during testing was described in just three studies (9.4%) (Table 2).

The reporting practices differed considerably with respect to study design. Among the studies examining reliability, the inclusion of rest periods between trials was reported in 15 studies (46.9%). In studies assessing validity, upper-limb restrictions were described in one study (20.0%), barefoot testing in two studies (40.0%), predefined error criteria for failed trials in three studies (60.0%), and the use of a YBT kit in three studies (60.0%). Among the studies that evaluated both reliability and validity, explicit specification of the trial order for each reach direction was reported in only one study (16.7%).

Fourteen studies (43.8%) reported the use of six practice trials for the YBT, whereas eight studies (25.0%) did not specify the number of practice trials performed. With regard to test trials, the majority of the studies (21 studies; 62.6%) employed three trials. Concerning rest periods, seven studies (21.9%) reported the implementation of breaks between reach directions, with substantial variability in rest duration ranging from 20 s to 5 min.

When stratified by participant attributes, studies involving injury populations demonstrated distinct procedural characteristics compared to those involving healthy controls and athletic populations. Within the injury group, the presence of practice trials was reported in six studies (85.7%), predefined error criteria for failed trials were described in six studies (85.7%), and the inclusion of rest periods between trials was reported in six studies (85.7%). Upper-limb restrictions were documented in two studies (28.6%), permission to lift the heel off the ground was not reported in any study (0.0%), and the explicit specification of the trial order for each reach direction was reported in only one study (14.3%) (Table 3).

## 4. Discussion

### 4.1. Reporting Heterogeneity of YBT Measurement Procedures

This scoping review examined the reporting frequency of participant characteristics and measurement procedures in studies assessing the reliability and validity of the YBT. A total of 32 studies were included and classified according to participant attributes and study design. Previous reviews have primarily focused on reliability coefficients or sports-specific performance characteristics of the YBT. In contrast, the present review examined how YBT measurement procedures were reported within reliability and validity research. The findings demonstrated marked heterogeneity in the reporting of YBT measurement procedures according to participant characteristics and research design. Several procedural elements showed distinct patterns, including upper-limb restrictions, heel lift allowance, and trial order specification. Importantly, this is the first scoping review to systematically map heterogeneity in the reporting of YBT measurement procedures within reliability and validity research.

Among the 32 eligible studies, 24 (75.0%) reported whether practice trials had been conducted. Considerable variability was observed in the number of practice trials, with six trials reported most frequently, followed by three and four trials. Plisky et al. described a protocol incorporating six practice trials for the traditional YBT [4], whereas Takahashi et al. reported that YBT performance values plateaued after three actual test trials when no prior practice trials were conducted [5]. These findings indicate substantial heterogeneity in the number of practice trials employed across studies. Given the relationship between practice trials and reliability outcomes, explicit reporting of whether practice trials were performed is important. With respect to other procedural elements, normalization of measured values was reported in 25 studies (78.1%), heel lift allowance in three studies (9.4%), and implementation of a pre-measurement warm-up in four studies (12.5%). In the original YBT protocol proposed by Plisky et al., only heel lift allowance was explicitly documented [7]. However, subsequent studies reported additional procedural details, including normalization to lower-limb length [4], heel lift allowance, and warm-up protocols [11]. Overall, inconsistent reporting of YBT measurement procedures may limit the interpretability and comparability of findings. Researchers and clinicians should therefore carefully verify YBT procedures while considering the characteristics of the populations being assessed.

### 4.2. Influence of Study Design on Reporting Practices

In the present review, the reporting frequency of the YBT measurement procedures differed according to the study design. Among the studies that exclusively assessed reliability, only six (28.6%) reported whether rest periods were incorporated. Previous research has recommended the inclusion of rest periods between practice and test trials, as well as within the YBT protocol itself, and has demonstrated that fatigue can adversely influence the reliability of measurement outcomes [8,40]. These findings suggest that the importance of reporting both the presence and duration of rest periods during YBT assessments has not been sufficiently emphasized in the existing literature. Huang et al. [6] noted that appropriate rest intervals may vary with participants’ muscle strength and endurance, complicating standardized recommendations. Nevertheless, failure to report rest periods that may affect measurement outcomes may introduce bias into the findings. In particular, reliability studies aimed at evaluating measurement properties should provide detailed descriptions of the testing procedures, including rest periods. In contrast, among the studies that evaluated validity only, reporting rates for upper-limb restrictions, barefoot testing, predefined error criteria for failed trials, and the use of a YBT kit were 20.0%, 40.0%, 60.0%, and 60.0%, respectively. These rates are lower than those observed in reliability focused studies. Accordingly, the findings of this review indicate systematic differences in the reporting frequency of YBT measurement procedures depending on study design. One possible explanation is that validity focused studies tend to prioritize associations between YBT outcomes and external criteria, whereas reliability studies inherently require more explicit standardization and documentation of testing protocols. This pattern, observed across multiple procedural elements, may reflect limitations in reporting quality in validity-oriented studies. Although the present review cannot determine whether these differences are directly attributable to study design, comprehensive reporting of measurement procedures should be ensured regardless of research purpose. This may also help explain why only one study (16.7%) specified the order of trials for each reach direction among studies assessing both reliability and validity.

### 4.3. Reporting Challenges in Clinical and Injury Populations

Finally, reporting patterns were examined according to participants’ attributes. Studies involving diseased or injured populations tended to exhibit lower reporting frequencies than the other two groups across multiple procedural elements, including the presence of practice trials, upper-limb restrictions, barefoot testing, allowance of heel lift, specification of trial order for each reach direction, predefined error criteria for failed trials, use of a YBT kit, and normalization of measured values. The comparatively lower reporting frequency observed in studies of diseased populations may be partially attributable to the task demands of the YBT. Previous research has demonstrated that restrictions on upper-limb movement and heel lift can influence balance control [41]. Furthermore, in individuals with lower-limb injuries, certain movement patterns such as those associated with inversion ankle sprains and anterior cruciate ligament injuries pose an elevated risk [42,43]. Although this study cannot fully explain these findings, given these condition-specific considerations, it is possible that some degree of flexibility in measurement procedures was applied when assessing participants in disease or injury groups. However, the included studies rarely reliability.

### 4.4. The Conceptual Importance of Key Procedural Elements

Regarding the number of practice trials, previous studies have reported variations across protocols. For example, whereas Alshehre et al. conducted a validity study involving six practice trials in patients with chronic low back pain, Scinicarelli et al. conducted a reliability study using two practice trials [26,27]. Based on these findings, when referencing studies on reliability and validity, it is important to consider participant characteristics (e.g., age, sex, and competitive level) and to systematically evaluate measurement procedures in populations with similar attributes. Furthermore, consulting prior research may aid in selecting an appropriate number of practice and test trials.

Furthermore, measurement conditions such as heel lift allowance and upper-limb restraint are critical procedural elements [44,45]. These conditions are related to the selection of outcome measures (e.g., maximum vs. mean values) and may influence the interpretation of results. Therefore, researchers should select measurement conditions and analytical methods in accordance with the study objectives and with consideration of comparability with the existing literature. In summary, clear and comprehensive reporting of the YBT measurement procedure may enhance comparability across studies and improve the applicability of this test across different populations.

### 4.5. Limitations

This study had several limitations. First, as the primary objective of this scoping review was to characterize the distribution of reported measurement procedures in studies assessing the reliability and/or validity of the YBT, causal relationships could not be established. Additionally, the magnitudes of the reported reliability and validity indices were not examined. Second, the participant attributes were categorized into broad groups (e.g., healthy, athletic, diseased, or injured populations), which may encompass substantial heterogeneity within each category. Accordingly, differences in reporting frequency should be interpreted as group-level rather than population-specific. Furthermore, studies were not excluded based on the presence or absence of descriptions of measurement procedures. In contrast, reporting of demographic information (e.g., participant characteristics) was required. Consequently, studies with insufficient demographic reporting may have been excluded, potentially leading to an overestimation of reporting frequency. Moreover, as this was a scoping review, no formal quality assessment was conducted; therefore, the findings should be interpreted with caution. Third, studies for which full-text articles were unavailable were excluded. Nevertheless, comprehensive search strategies were implemented, including the absence of publication year restrictions and the use of broad and relevant search terms to maximize the coverage of the existing literature. Moreover, the review was conducted in accordance with established methodological recommendations to ensure methodological rigor. Finally, temporal trends in the reporting of YBT measurement procedures were not evaluated. Future investigations examining changes in reporting practices over time may yield further insights into the evolution and standardization of YBT measurement protocols.

## 5. Conclusions

This scoping review examined YBT measurement procedures in studies evaluating reliability and/or validity and systematically synthesized the extent to which these procedures were reported. Overall, substantial heterogeneity was identified in the reporting of YBT measurement procedures across studies, underscoring the need for greater consistency and transparency in future reliability and validity studies. The findings demonstrate that YBT measurement procedures vary according to study design and participant characteristics. Rather than advocating uniform measurement protocols, these results highlight the importance of improving the standardization of reporting practices to enhance interpretability and cross-study comparability. Moreover, the results underscore the need for further research aimed at enhancing the quality, transparency, and consistency of the reporting of YBT measurement procedures. Future studies should explicitly report key procedural elements, such as practice trials, rest periods, heel lift allowance, and trial order, to facilitate meaningful comparisons across studies.

## Figures and Tables

**Table 1:** Characteristics of the Included Studies (n = 32).

Author (Year)	Study Focus	Country	Participants (as Reported)	Population Group	Sex	Sample Size	Age (Years)
Plisky (2009) [4]	Reliability	USA	Athletes	Athletic	Male	n = 15	19.7 ± 0.8
Shaffer (2013) [3]	Reliability	USA	Military personnel	Healthy	Male/ Female	n = 64 (male: 53; female: 11)	25.2 ± 3.8
Faigenbaum (2014) [12]	Reliability	USA	Healthy children	Healthy	Male/ Female	n = 188	6.9–12.1
Almeida (2017) [13]	Reliability & Validity	Brazil	Healthy recreational adults/Injury to the lower limbs	Injury- related	Male/ Female	n = 40	20.9 ± 2.5
Walbright (2017) [14]	Validity	USA	Basketball, volleyball athletes	Athletic	Female	n = 36	Not reported
Linek (2017) [15]	Reliability	Poland	Soccer players	Athletic	Male	n = 38	15.6
Hoch (2017) [16]	Reliability	USA	Ice hockey players	Athletic	Female	n = 20	19.6 ± 1.3
Kenny (2018) [17]	Reliability	Canada	Dancers	Athletic	Male/ Female	n = 38 (male: 3; female: 35)	male: 16.6; female: 19.2
Smith (2018) [18]	Reliability	USA	Soccer, baseball, lacrosse, softball, rugby	Athletic	Male/ Female	n = 110 (male: 51; female: 59)	male: 15.9 ± 1.2; female: 16.1 ± 1.2
Greenberg (2019) [7]	Reliability	USA	Athletes (sport not specified)	Athletic	Female	n = 25	12.7 ± 0.6
Lacey (2019) [19]	Reliability & Validity	Ireland	Gaelic football, soccer, rugby athletes	Athletic	Male/ Female	n = 19	male: 24.0 ± 4.0; female: 28.0 ± 4.0
Freund (2019) [20]	Reliability & Validity	USA	Healthy older adults	Healthy	Female	n = 86	50–59: 55.7 ± 2.8; 60–69: 63.6 ± 2.8; 70–79: 73.6 ± 3.0
Sipe (2019) [21]	Reliability	USA	Healthy older adults	Healthy	Male/ Female	n = 30 (male: 15; female: 15)	66.8 ± 5.4
Schwiertz (2019) [22]	Reliability	Germany	Healthy adolescents	Healthy	Male/ Female	n = 221	Grade 6: 11.6 ± 0.5; Grade 7: 12.4 ± 0.5; Grade 8: 13.3 ± 0.5; Grade 9: 14.3 ± 0.5; Grade 10: 15.3 ± 0.6; Grade 11: 16.4 ± 0.5
Schwiertz (2020) [23]	Validity	Germany	Soccer and swimming athletes	Athletic	Male	n = 212 (soccer: 138; swimming: 74)	soccer: 14.4 ± 1.9; swimming: 12.3 ± 2.1
Jagger (2020) [24]	Reliability & Validity	USA	Healthy adults	Healthy	Male/ Female	n = 28 (male: 17; female: 11)	25.0 ± 2.2
Grazette (2020) [25]	Reliability	UK	Soccer players	Athletic	Male	n = 56	14.0 ± 1.1
Scinicarelli (2021) [26]	Reliability	Germany	Healthy adults	Healthy	Male/ Female	n = 22 (male: 14; female: 8)	23.3 ± 3.9
Alshehre (2021) [27]	Reliability & Validity	USA	Low back pain	Injury- related	Male/ Female	n = 30	27.4 ± 4.9
Iglesias-Caamaño (2022) [28]	Reliability	Spain	Professional volleyball players	Athletic	Male	n = 13	23.3 ± 4.2
Stoddard (2022) [29]	Reliability	USA	Healthy adolescents	Healthy	Male/ Female	n = 26 (male: 4; female: 22)	13.6 ± 1.0
Salas-Gómez (2022) [30]	Validity	Spain	Ankle fracture	Injury- related	Male/ Female	n = 22 (male: 12; female: 9)	43.5 ± 10.2
Kuniki (2022) [31]	Validity	Japan	Healthy adults	Healthy	Male/ Female	n = 51	20–24
Jouira (2022) [32]	Reliability	Tunisia	Athletes (sport not specified)	Athletic	Male	n = 13	25.1 ± 4.5
Kattilakoski (2023) [33]	Reliability	Finland	Healthy recreational adults	Healthy	Male/ Female	n = 16 (male: 4; female: 12)	39.1 ± 6.8
Foldager (2023) [34]	Reliability	Denmark	Healthy recreational adults	Healthy	Male/ Female	n = 51 (male: 30; female: 21)	28.0 ± 7.2
Ünal (2024) [35]	Reliability & Validity	Turkey	Hearing-impaired athletes	Injury- related	Not reported	n = 60	18–21
Zheng (2024) [36]	Reliability	China	Flatfoot	Injury- related	Male/ Female	n = 30 (male: 14; female: 16)	21.5 ± 0.3
Miralles (2024) [37]	Reliability	Spain	Healthy adults	Healthy	Male	n = 63	23.5 ± 4.5
Takahashi (2025) [5]	Reliability	Japan	Soccer players	Athletic	Female	n = 31	16.5 ± 0.5
Doran (2025) [38]	Reliability	USA	Post-ACL reconstruction	Injury- related	Male/ Female	n = 30	19.9 ± 7.7
Rungruangbaiyok (2025) [39]	Validity	Thailand	Locomotive syndrome	Injury- related	Not reported	n = 60	67.2 ± 4.6

Abbreviations: ACL, anterior cruciate ligament.

**Table 2:** Overall Reporting Frequency of Y Balance Test Measurement Procedures Across Included Studies (n = 32).

Measurement Procedure	Studies Reporting the Procedure, n (%)	Reliability Studies (n = 21)	Validity Studies (n = 5)	Reliability & Validity Studies (n = 6)
Practice trials reported	24 (75.0%)	14 (66.7%)	4 (80.0%)	6 (100.0%)
Predefined error criteria for failed trials	25 (78.1%)	16 (76.2%)	3 (60.0%)	6 (100.0%)
Use of a YBT kit	23 (71.9%)	16 (76.2%)	3 (60.0%)	4 (66.7%)
Normalization of outcome measures	25 (78.1%)	17 (81.0%)	4 (80.0%)	4 (66.7%)
Pre-measurement warm-up	4 (12.5%)	3 (14.3%)	1 (20.0%)	0 (0.0%)
Heel lift allowance	3 (9.4%)	2 (9.5%)	1 (20.0%)	0 (0.0%)
Rest periods between trials or reach directions	15 (46.9%)	6 (28.6%)	3 (60.0%)	6 (100.0%)
Upper-limb restrictions	11 (34.4%)	7 (33.3%)	1 (20.0%)	3 (50.0%)
Specification of trial order for each reach direction	14 (43.8%)	11 (52.4%)	2 (40.0%)	1 (16.7%)
Barefoot testing	18 (56.3%)	11 (52.4%)	2 (40.0%)	5 (83.3%)

**Table 3:** Reporting Frequency of Y Balance Test Measurement Procedures Stratified by Participant Group.

Measurement Procedure	Healthy Group (n = 12)	Athletic Group (n = 13)	Injury-Related Group (n = 7)
Practice trials reported	9 (75.0%)	9 (69.2%)	6 (85.7%)
Rest periods between trials or reach directions	3 (25.0%)	6 (46.2%)	6 (85.7%)
Upper-limb restrictions	6 (50.0%)	3 (23.1%)	2 (28.6%)
Heel lift allowance	3 (25.0%)	0 (0.0%)	0 (0.0%)
Specification of trial order for each reach direction	5 (41.7%)	8 (61.5%)	1 (14.3%)
Predefined error criteria for failed trials	11 (91.7%)	8 (61.5%)	6 (85.7%)
Use of a YBT kit	9 (75.0%)	10 (76.9%)	4 (57.1%)
Normalization of outcome measures	11 (91.7%)	10 (76.9%)	4 (57.1%)
Barefoot testing	7 (58.3%)	7 (53.8%)	4 (57.1%)

Abbreviations: YBT, Y Balance Test.

## Data Availability

Data is contained within the article.

## Supplementary Materials

The following supporting information can be downloaded at: https://www.mdpi.com/article/10.3390/sports14050191/s1, Table S1. Preferred Reporting Items for Systematic reviews and Meta-Analyses extension for Scoping Reviews Checklist; Table S2. Complete search strategies for each database.
