# Supplementary material for: Reporting of Y Balance Test Measurement Procedures in Reliability and Validity Studies: A Scoping Review
Source: Sports (Basel). 2026 May 6;14(5):191. doi: 10.3390/sports14050191 (PMC13210642; doi:10.3390/sports14050191)
Supplement: Supplementary file 1 [file sports-14-00191-s001.zip › Supplementary Material 2.pdf]

Table S2. Complete search strategies for each database.

pubmed

("Y balance test"[Title/Abstract] OR "Y-balance test"[Title/Abstract] OR "Lower Quarter Y Balance Test"[Title/Abstract] OR YBT[Title/Abstract] OR "Y-BT"[Title/Abstract] OR "Y Balance"[Title/Abstract] OR "Star Excursion Balance Test"[Title/Abstract] OR SEBT[Title/Abstract] OR "modified Star Excursion Balance Test"[Title/Abstract] OR mSEBT[Title/Abstract] OR m-SEBT[Title/Abstract]) AND (reliability[Title/Abstract] OR reproducibility[Title/Abstract] OR "test-retest"[Title/Abstract] OR "intra-rater"[Title/Abstract] OR "inter-rater"[Title/Abstract] OR "measurement error"[Title/Abstract] OR "intraclass correlation"[Title/Abstract] OR ICC[Title/Abstract] OR psychometrics[MeSH Terms] OR "reproducibility of results"[MeSH Terms] OR validity[Title/Abstract] OR "construct validity"[Title/Abstract] OR "criterion validity"[Title/Abstract] OR "concurrent validity"[Title/Abstract] OR "predictive validity"[Title/Abstract] OR "discriminant validity"[Title/Abstract] OR "measurement properties"[Title/Abstract])

Cochrane

("Y balance test":ti,ab OR "Y-balance test":ti,ab OR "Lower Quarter Y Balance Test":ti,ab OR YBT:ti,ab OR Y-BT:ti,ab OR "Y Balance":ti,ab OR "Star Excursion Balance Test":ti,ab OR SEBT:ti,ab OR "modified Star Excursion Balance Test":ti,ab OR mSEBT:ti,ab OR m-SEBT:ti,ab) AND (reliability:ti,ab OR reproducibility:ti,ab OR test-retest:ti,ab OR intra-rater:ti,ab OR inter-rater:ti,ab OR "measurement error":ti,ab OR "intraclass correlation":ti,ab OR ICC:ti,ab OR [mh psychometrics] OR [mh "reproducibility of results"] OR validity:ti,ab OR "construct validity":ti,ab OR "criterion validity":ti,ab OR "concurrent validity":ti,ab OR "predictive validity":ti,ab OR "discriminant validity":ti,ab OR "measurement properties":ti,ab)

CINAHL

((TI "Y balance test" OR AB "Y balance test") OR (TI "Y-balance test" OR AB "Y-balance test") OR (TI "Lower Quarter Y Balance Test" OR AB "Lower Quarter Y Balance Test") OR (TI YBT OR AB YBT) OR (TI Y-BT OR AB Y-BT) OR (TI "Y Balance" OR AB "Y Balance") OR (TI "Star Excursion Balance Test" OR AB "Star Excursion Balance Test") OR (TI SEBT OR AB SEBT) OR (TI "modified Star Excursion Balance Test" OR AB "modified Star Excursion Balance Test") OR (TI mSEBT OR AB mSEBT) OR (TI m-SEBT OR AB m-

SEBT)) AND ((TI reliability OR AB reliability) OR (TI reproducibility OR AB reproducibil ity) OR (TI test-retest OR AB test-retest) OR (TI intra-rater OR AB intra-rater) OR (TI inter-rater OR AB inter-rater) OR (TI "measurement error" OR AB "measurement error") OR (TI "intraclass correlation" OR AB "intraclass correlation") OR (TI ICC OR AB ICC) OR (MH psychometrics+) OR (MH "reproducibilit y of results+") OR (TI validity OR AB validity) OR (TI "construct validity" OR AB "construct validity") OR (TI "criterion validity" OR AB "criterion validity") OR (TI "concurrent validity" OR AB "concurrent validity") OR (TI "predictive validity" OR AB "predictive validity") OR (TI "discriminant validity" OR AB "discriminant validity") OR (TI "measurement properties" OR AB "measurement properties"))

WHO-ICTRP search strategy

Condition: “Y balance test” OR “Y-balance test” OR YBT

Recruitment status: ALL

ClinicalTraials.gov search strategy

Condition/disease: “Y balance test” OR “Y-balance test” OR YBT
